# Buruli Ulcer in South Western Nigeria: A Retrospective Cohort Study of Patients Treated in Benin

**DOI:** 10.1371/journal.pntd.0003443

**Published:** 2015-01-08

**Authors:** Estelle Marion, Kevin Carolan, Ambroise Adeye, Marie Kempf, Annick Chauty, Laurent Marsollier

**Affiliations:** 1 Centre de Diagnostic et de Traitement de l'Ulcère de Buruli de Pobè, Fondation Raoul Follereau, Pobè, Bénin; 2 ATOMycA, Inserm Atip-Avenir Team, CRCNA, Inserm U892, 6299 CNRS, University and Centre Hospitalier Universitaire d'Angers, Angers, France; 3 UMR Mivegec, IRD-CNRS-Universities of Montpellier I and II, Montpellier, Centre IRD de Montpellier, Montpellier, France; 4 Laboratoire de bactériologie et d'hygiène hospitalière, Centre Hospitalier Universitaire d'Angers, Angers, France; Fondation Raoul Follereau, France

## Abstract

Nigeria is known to be endemic to Buruli ulcer, but epidemiological data are remarkably rare. Here, we present a large cohort of 127 PCR-confirmed *M. ulcerans* infection patients coming from Nigeria and treated in a neighbouring country, Benin. Severe lesions and delay of consultation are factors that should encourage establishment of a treatment centre in South Western Nigeria.

## Author Summary

Buruli ulcer is known to be endemic to Nigeria since at least 1967, however epidemiological data are rare and incomplete. In total, only 51 Buruli ulcer patients were described in 45 years, all found in Southern Nigeria. This is likely a result of the lack of adequate public health structures dedicated to the diagnosis and treatment of Buruli ulcer in the region. Here, we report a large cohort of 127 PCR-confirmed Nigerian patients treated in neighbouring Benin. Nigerian patients presented mainly severe lesions of Buruli ulcer, and this is linked by the fact that period prior consultation is delayed (24% of the patients waited more than one year between the beginning of the lesion and the consultation in the Buruli ulcer treatment centre in Pobè). We identify South Western Nigeria as an important endemic area for Buruli ulcer, and believe our results will be of importance to Nigerian health authorities, the World Health Organisation and NGO's involved in management of Buruli ulcer.

## Introduction

Buruli ulcer, the third most common mycobacterial disease of humans after tuberculosis and leprosy, is an important disfiguring and disabling cutaneous infection caused by *Mycobacterium ulcerans*. Buruli ulcer was declared an emerging skin disease of public health concern by the World Health Organization (WHO) in 1998. Epidemiological studies show that this mycobacteriosis is most common in populations living near rivers, swamps and wetlands [1], [2], [3], while the role of aquatic insects or mosquitoes as reservoirs and vectors of *M. ulcerans* has been proposed but remains controversial [4], [5], [6], [7], [8], [9], [10]. Since 2004, the WHO has recommended a daily treatment of rifampin and streptomycin for 8 weeks as a first line treatment [11]. In addition to the antibiotherapy, surgery is often necessary on large lesions to remove necrotic tissues before carrying out a skin graft. Treatments and wound care need to be done in competent and specialized health structures, which are uncommon in the most Buruli ulcer endemic countries. The disease is widespread in West and Central Africa, French Guiana and much of South America, and Australia, and most of the burden of the disease falls on West and Central Africa [12], with the majority of cases being in Cote d'Ivoire. In the available epidemiological data, Nigeria presents a notable gap in cases, surrounding countries such as Cameroon and Benin which have highly endemic regions, and this gap is likely due to underreporting and the lack of adequate public health structures [3], [13].

**Figure 1:**
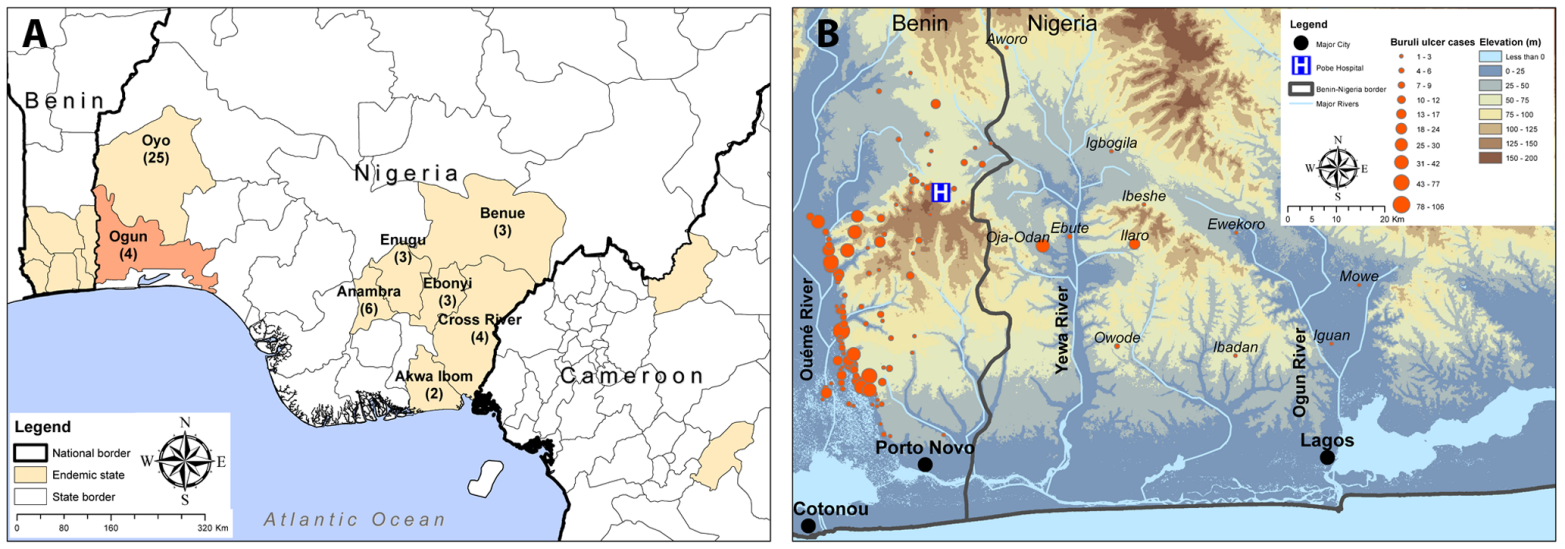
Localisation of Buruli ulcer patients in Nigeria. (A) Nigerian districts where Buruli ulcer patients were already described at least once, and neighbouring countries areas where Buruli ulcer is endemic. The number of cases described since 1967 is indicated for each Nigerian district. (B) Location of Benin and Nigerian patients coming in CDTUB-Pobè for treatment of Buruli ulcer.

Buruli ulcer was first described in Nigeria in 4 patients living in the Benue State in 1967 [14] (Fig. 1A). In 1976 a total of 24 Buruli ulcer patients were described in an area around Ibadan town, Oyo State [15] (Fig. 1A). Thirty years later the Nigerian government, with the support of the WHO, conducted an assessment of Buruli ulcer presence in the Southern and Southeastern states of the country [16], where cases had been previously reported. As a result of that study, 14 patients were considered likely to have a Buruli ulcer, and came from 5 different states: Anambra, Cross river, Enugu, Ebonyi and Akwa Ibom [16] (Fig. 1A). Most recently, the presence of Buruli ulcer in Nigeria was mentioned in a manuscript in which 9 *M. ulcerans* strains were isolated from patients living in Oyo, Anambra, Cross river Enugu, Ebonyi or Ogun states, between year 2006 and 2012 [17] (Fig. 1A). In total, 51 Buruli ulcer patients were described in 45 years, all found in Southern Nigeria. This region is characterised by a tropical rainforest climate, similar to Buruli ulcer endemic areas around the Gulf of Guinea. The Northern part of the Nigeria is associated with a tropical dry climate which is not known to be associated with Buruli ulcer presence.

Here, we present a large cohort of 127 PCR-confirmed *M. ulcerans* infection patients coming from South Western Nigeria and treated in a neighbouring country, Benin, between the years of 2004 and 2013.

## Materials and Methods

### Buruli ulcer case data

This retrospective study was conducted in the Centre de Diagnostic et de Traitement de l'Ulcère de Buruli in Pobè, Benin. This structure, built in 2004, is one of the four medical centers dedicated to the diagnosis and treatment of Buruli ulcer present in Benin and the closest to the border with Nigeria [18], [19]. In the Buruli ulcer registry of the medical centre, we identified all patients with PCR-confirmed *M. ulcerans* infection and Nigerian origin diagnosed in CDTUB of Pobè from 2004 to 2013. We recorded detailed patient data regarding demographic characteristics, medical history and clinical features. The Nigerian origin was defined as a patient living in Nigeria, ie working in Nigeria for an adult or going to school in Nigeria for a child for at least two years. We considered therefore that the contamination of the patient occurred the most likely in Nigeria. In the case of no or unclear information about the origin of the patient in the medical record, the patient was not included in the present cohort to avoid a potential bias.

### Ethics statement

Access to the registry was approved by the institutional review board of the CDTUB and the national Buruli ulcer control authorities. Case data used in the study were anonymised.

### Statistical analysis

Continuous variables were summarized using median and categorical variables were summarized using frequencies. Comparison of variables was done using a Mann-Whitney test.

## Results and Discussion

**Figure 2:**
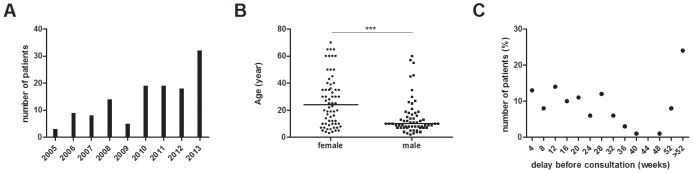
Buruli ulcer Nigerian patients. (A) Number of new Buruli ulcer patients coming from Nigeria and treated in Buruli ulcer treatment centre of Pobè, Benin between 2005 and 2013. (B) Age of patients by gender, *** *P* = 0.0001. (C) Distribution of patient consultation delay.

**Figure 3:**
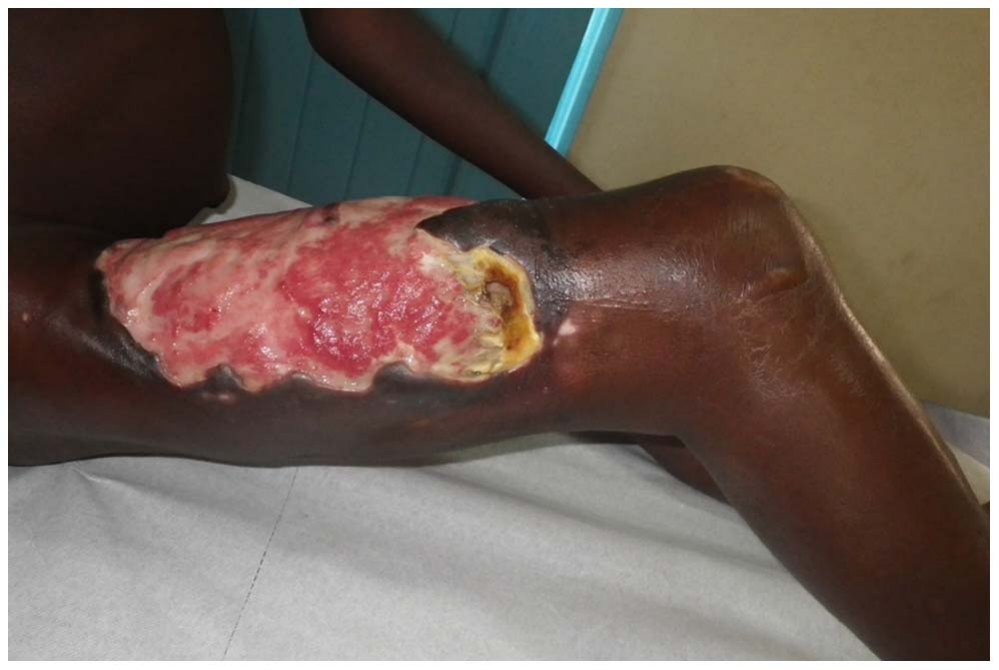
A typical category III lesion of Buruli ulcer. Most Nigerian patients presented in medical center of Pobè in Benin with extensive ulcerative lesions. On the picture, the lesion of a Nigerian 10 years old child on the right thigh and with the typical characteristic of a late stage of the disease: large painless ulceration with presence of necrosis and undermined edges.

We have collected data of Nigerian patients that have been treated in the Buruli ulcer treatment centre of Pobè, Benin, which is located at 3 kilometres from the border with Ogun State, Nigeria. In 2005 the first Nigerian came to Pobè treatment centre with a Buruli ulcer lesion, one year after the building of the hospital. From 2005 to 2013, 127 Nigerian patients were diagnosed and treated for Buruli ulcer in this hospital and the number of new cases is increasing each year (Fig. 2A). Clinico-epidemiological features of the patients are presented in the table (Table 1). There were 52% (66) females and 48% (61) males with a median age of 24 and 10 years, respectively (Fig. 2B). Such sex ratio variation with age at diagnosis has been previously reported and could be due to differential exposure to *M. ulcerans*, or different responses by the family and community to infection in boys versus girls [1], [13], [18]. The WHO categorises lesions by size in three categories: category I, maximum diameter <5 cm, category II, 5–15 cm, and category III, >15 cm. Nigerian Patients commonly present with large lesions, with 60% (73) of category III, including 20% with osteomyelitis (Fig. 3). There were 8% (10) category I and 36% (42) category II. 78% (99) of lesions were at the ulcerative stage. These data show that Nigerian patients present mainly late stages of Buruli ulcer. This is confirmed by the delay before consultation, where 24% of the patients had waited more than one year between the beginning of the lesion and the consultation in the Buruli ulcer treatment centre in Pobè (Fig. 2C). By comparison, among all the Buruli ulcer patients treated for Buruli ulcer between 2005 and 2011 in Pobè treatment center, lesions of category 3 represented only 36% of the lesions [18].

**Table 1:** Clinico-epidemiological features of Buruli ulcer patients from Nigeria.

Characteristic		n	%
Gender	male	61	48%
	female	66	52%
Age	≤15	68	53,5%
	>15	59	46,5%
Category of lesions*	I	10	8%
	II	42	33,6%
	III	73	58,4%
Clinical forms	ulcerated simple	34	26,8%
	ulcerated mixed	65	51,2%
	non ulcerated	28	22%
Site of lesions	lower limb	72	56,7%
	upper limb	36	28,3%
	other	7	5,5%
	disseminated	12	9,45%

*no available for 2 patients.

We were able to georeference the origin of 68% (86) of Nigerian patients. They came mainly from the Ogun state which shares its western border with Benin (Fig. 1B). The furthest village, Mowe, is about 100 km from the Buruli ulcer treatment center, and it takes around 6 hours to reach Pobè by motobike. The Nigerian villages reporting the most Buruli ulcer patients are those closest to Pobè: Oja-Odan, Ebute, Ilaro. For 32% of Nigerian patients, the name of the Nigerian locality, recorded in their medical folder, does not match with a name of a Nigerian village administratively known.

The Nigerian endemic area in Ogun state is divided in two drainage basins, the Yewa and the Ogun rivers, while Ouémé/Plateau endemic department in Benin is traversed by the Ouémé river. These 3 draining systems discharge in separate lagoons that are interconnected by channels at the delta. Southeast Benin and Southwest Nigeria do not belong to the same drainage system, but these two Buruli ulcer endemic areas have a similar topography and climate, characterised by tropical rainforest, changeable topography with many small hills and fertile plains. Similar environments are encountered in other Buruli ulcer endemic areas of West Africa, for which the patients are found around different drainage systems but always with broad fertile richly inundates plains [17].

This report is the first description of a large cohort of PCR-confirmed Buruli ulcer patients coming from South Western Nigeria. As no health centre is dedicated to diagnose and treat Buruli ulcer in this country, patients close to the south western border turn to the Beninese health system, where specialised facilities treat Buruli ulcer free of charge or with a small participation. This report supports the establishment of a Buruli ulcer surveillance system in Nigeria, encouraging the training of health workers in Ogun state, and promotes the establishment of a Buruli ulcer treatment centre in South Western Nigeria.

## Supporting Information

S1 Checklist **STROBE Checklist.**
(DOC)
