# Supplementary material for: Buruli Ulcer in South Western Nigeria: A Retrospective Cohort Study of Patients Treated in Benin
Source: PLoS Negl Trop Dis. 2015 Jan 8;9(1):e3443. doi: 10.1371/journal.pntd.0003443 (PMC4287556; doi:10.1371/journal.pntd.0003443)
Supplement: S1 Checklist — STROBE Checklist. (DOC) [file pntd.0003443.s001.doc]

STROBE Statement—Checklist of items that should be included in reports of ***cohort studies***

|  |  | Recommendation |
| --- | --- | --- |
| **Title and abstract** | √  √ | (*a*) Indicate the study’s design with a commonly used term in the title or the abstract |
|  |  | (*b*) Provide in the abstract an informative and balanced summary of what was done and what was found |
| Introduction | | |
| Background/rationale | √ | Explain the scientific background and rationale for the investigation being reported |
| Objectives | √ | State specific objectives, including any prespecified hypotheses |
| Methods | | |
| Study design | √ | Present key elements of study design early in the paper |
| Setting | √ | Describe the setting, locations, and relevant dates, including periods of recruitment, exposure, follow-up, and data collection |
| Participants | √ | (*a*) Give the eligibility criteria, and the sources and methods of selection of participants. Describe methods of follow-up |
|  |  | (*b*) For matched studies, give matching criteria and number of exposed and unexposed |
| Variables | √ | Clearly define all outcomes, exposures, predictors, potential confounders, and effect modifiers. Give diagnostic criteria, if applicable |
| Data sources/ measurement | √ | For each variable of interest, give sources of data and details of methods of assessment (measurement). Describe comparability of assessment methods if there is more than one group |
| Bias | √ | Describe any efforts to address potential sources of bias |
| Study size | √ | Explain how the study size was arrived at |
| Quantitative variables | √ | Explain how quantitative variables were handled in the analyses. If applicable, describe which groupings were chosen and why |
| Statistical methods | √ | (*a*) Describe all statistical methods, including those used to control for confounding |
|  |  | (*b*) Describe any methods used to examine subgroups and interactions |
|  |  | (*c*) Explain how missing data were addressed |
|  |  | (*d*) If applicable, explain how loss to follow-up was addressed |
|  |  | (*e*) Describe any sensitivity analyses |
| Results | | |
| Participants | √ | (a) Report numbers of individuals at each stage of study—eg numbers potentially eligible, examined for eligibility, confirmed eligible, included in the study, completing follow-up, and analysed |
|  |  | (b) Give reasons for non-participation at each stage |
|  |  | (c) Consider use of a flow diagram |
| Descriptive data | √ | (a) Give characteristics of study participants (eg demographic, clinical, social) and information on exposures and potential confounders |
|  |  | (b) Indicate number of participants with missing data for each variable of interest |
|  |  | (c) Summarise follow-up time (eg, average and total amount) |
| Outcome data | √ | Report numbers of outcome events or summary measures over time |
| Main results | √ | (*a*) Give unadjusted estimates and, if applicable, confounder-adjusted estimates and their precision (eg, 95% confidence interval). Make clear which confounders were adjusted for and why they were included |
|  |  | (*b*) Report category boundaries when continuous variables were categorized |
|  |  | (*c*) If relevant, consider translating estimates of relative risk into absolute risk for a meaningful time period |
| Other analyses | √ | Report other analyses done—eg analyses of subgroups and interactions, and sensitivity analyses |
| Discussion | | |
| Key results | √ | Summarise key results with reference to study objectives |
| Limitations | √ | Discuss limitations of the study, taking into account sources of potential bias or imprecision. Discuss both direction and magnitude of any potential bias |
| Interpretation | √ | Give a cautious overall interpretation of results considering objectives, limitations, multiplicity of analyses, results from similar studies, and other relevant evidence |
| Generalisability | √ | Discuss the generalisability (external validity) of the study results |
| Other information | | |
| Funding | √ | Give the source of funding and the role of the funders for the present study and, if applicable, for the original study on which the present article is based |
